# Identification of pressed and extracted vegetable oils by headspace GC-MS

**DOI:** 10.1016/j.heliyon.2023.e18532

**Published:** 2023-07-21

**Authors:** Yang Liu, Zhenlin Chai, Yu Haixia

**Affiliations:** Zhejiang Academy of Forestry (Zhejiang Provincial Key Laboratory of Biological and Chemical Utilization of Forest Resources), 399^#^ Liuhe Road, Xihu District, Hangzhou, Zhejiang, 310023, China

**Keywords:** Headspace GC-MS, Pressed oil, Extraction oil, No. 6 solvent

## Abstract

Edible vegetable oils are produced either by mechanical pressing or extraction. Although pressing retains the inherent flavor and nutritional value of the oil, the oil yield is low and the process expensive. Extraction methods have high oil yields, low processing costs, and economic benefits; however, No. 6 solvent, which may pose potential risks to human health, is commonly used in the extraction and cleaning process. Differentiating extracted oil containing these solvents from pressed oil, for quality control, based on visual appearance is difficult. Hence, in this study, an identification method using the characteristic components of solvent No. 6 under optimized headspace Gas chromatography-mass spectrometry (GC-MS) conditions was established. It also provided a reference for quality control of industrial production by estimating the amount of solvent present in the oil. Results showed that, in addition to five main components (2-methylpentane, 3-methylpentane, and *n*-hexane, Methylcyclopentane, Cyclohexane), accounting for 97% of the solvent, No. 6 solvent also contains 16 types of organic substances, such as olefins, aromatic hydrocarbons, and polycyclic aromatic hydrocarbons. Under optimized headspace GC-MS conditions (headspace sampler equilibrium temperature = 150 °C), the No. 6 solvent exhibits high linearity over a concentration range of 0.05–1 mg/kg with a correlation coefficient of 0.999 and a detection limit of 0.01 mg/kg. Pressed and extracted oils can be determined as follows: If three or fewer main components of the No. 6 solvent are detected, and the total content of No. 6 solvent is less than 0.5 mg/kg, it is a pressed oil; if four or more main components of No. 6 solvent are detected, or the total content of No. 6 solvent is ≥0.5 mg/kg, it is confirmed as an extracted oil.

## 1 Introduction

Edible vegetable oils, such as peanut, sesame, and various nut oils, are traditionally produced by mechanical pressing, which is a relatively expensive process, and are commonly used as flavor oils [1]. Pressing oil retains the inherent flavor and nutritional value of the oil. However, the oil yield is low [2,3]. In addition to pressing, extraction is a major method for manufacturing edible vegetable oil. Extraction is based on the principle of similar phase solubility when extracted with organic solvents [4]; extraction methods have high oil yields, low processing costs, and economic benefits [5]. Compared with pressed vegetable oils, the oil yield of the extraction method reaches almost 100%; however, some nutrient elements in vegetables cannot be extracted because of their similar miscibility [6]. Currently, in industrialized countries, more than 90% of the total vegetable oil is produced by extracting methods (Hayyan et al*.*, 2017); [7]. Pressed oils are considerably more expensive than extracted oils; however, these two types of oils are difficult to distinguish visually. Few studies have distinguished between pressed and extracted oils. Therefore, a method that can effectively identify the process involved in vegetable oil production is required.

Moreover, edible oil is a basic component of every meal, therefore, the safety and quality of edible oils are major health concerns. [[8], [9], [10]]; (Pasri et al*.*, 2023). No. 6 solvent, commonly used for extraction and cleaning, can considerably increase oil yield and productivity. It is separated from raffinate oil (GB 16629–2008) and consists of a mixture of light alkanes, cycloalkanes, and other low-grade alkanes with hex alkanes (C6) as the main component [11,12]. Although it can increase yield, it may pose potential risks to human health. Organic solvents damage skin barrier function, peripheral nerves, and hematopoietic function [13]; Drabi et al*.*, 2022; [14]. No. 6 solvent, whose main component is hexane, remains in extracted oil. Because of its high-fat solubility, hexane can be easily accumulated in the human body and is toxic to the nervous system; moreover, it anesthetizes the respiratory center, and damages body oils [15]. The China National Standard (GB 5009.262–2016) has set the standard limit of solvent as 10 mg of extraction solvent per kg edible oil.

Many studies have been conducted on edible oil quality inspection using colorimetric sensors, Fourier Transform Infrared (FT-IR) spectroscopy, Nuclear Magnetic Resonance (NMR) spectroscopy, two-dimensional correlation spectroscopy, Matrix-Assisted Laser Desorption Ionization (MALDI) imaging mass spectrometry, and Gas Chromatography-Mass Spectrum (GC-MS) spectrometry [[16], [17], [18], [19]].

Gas chromatography (GC), one of the main methods for detecting solvent residues [20,21], is widely used in quality inspection of pharmaceuticals, leather, vegetable oil, and other products [[22], [23], [24]]. The detection limit of GC for solvent residues in vegetable oils is 2 mg/kg; however, false positivity is inevitable (GB 5009.262–2016). In addition to instrument false positivity, lubricants in squeezing machines containing the main components of the No. 6 solvent may contaminate vegetable oil during production [14]. Static headspace coupled with GC is an appropriate method for the analysis of solvent residues [25,26] as it is accurate with a lower detection limit than that of GC [13,14].

In this study, the feasibility of using headspace solid-phase microextraction techniques coupled with GC-MS for differentiating extracted vegetable oils from pressed vegetable oils was investigated. The characteristic components of the extraction solvent that may exist in the vegetable oil were analyzed and used to identify pressed oil.

This study aims to precisely identify pressed and extracted vegetable oils according to solvent residues and provide a reference for quality control of industrial production. Furthermore, it aims to facilitate accurate analysis and improvement of vegetable oil testing standard formulations.

## 2 Materials and methods

### 2.1 Vegetable oil

**Fig. 1:**
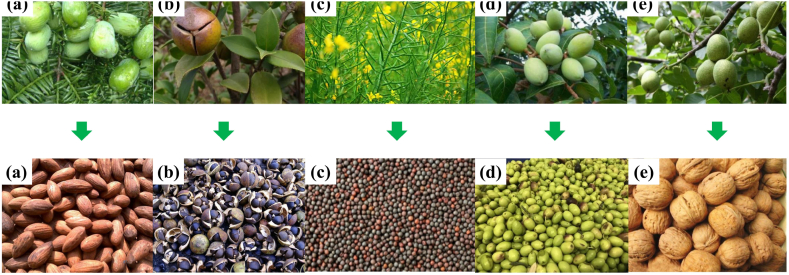
Oil seeds a: Chinese torreya; b: *Camellia oleifera* seed; c: Rapeseed; d: Olive; e: Chinese walnut.

*Camellia oleifera* seed oils: Pressed crude oils were produced in the laboratory (seeds were dried, roasted, crushed at 103 °C, and then pressed, oil was collected and filtered with an analytical filter paper) and extracted oils were purchased from a market oil; Chinese Torreya oils and Chinese walnut oils: Pressed crude oils were produced in the laboratory; Pressed oils provided by the companies included 10 types of rapeseed oil, six types of olive oil, six types of soybean oil, and eight types of camellia seed oil (Fig. 1(a–e)).

### 2.2 Reagent and solution

Solvent stock solution No. 6 (10 mg/ml) was purchased from the National Center for Standard Materials (No. cdhk-bw3599). Analytically pure *N*-dimethylacetamide was purchased from Shanghai Anpu Experimental Technology Co., Ltd.

### 2.3 Instruments and equipment

The instruments and equipment included an Agilent G1888 headspace sampler, an Agilent 7890A-5975B GC-MS meter, Agilent headspace injection bottles (20 ml) (at Zhejiang Provincial Key Laboratory of Biological and Chemical Utilization of Forest Resources, Hangzhou, Zhejiang, China), an Agilent bottleneck opener, an electronic balance (sensitivity 0.1 mg, Shanghai Mettler Company), a pipette (1 ml, 100 μl, 10 μl Eppendorf), an IKA SM3 vortex oscillator, and an oil press (zyj-9018 Best day).

### 2.4 Test methods

#### 2.4.1 Chromatographic conditions

**Fig. 2:**
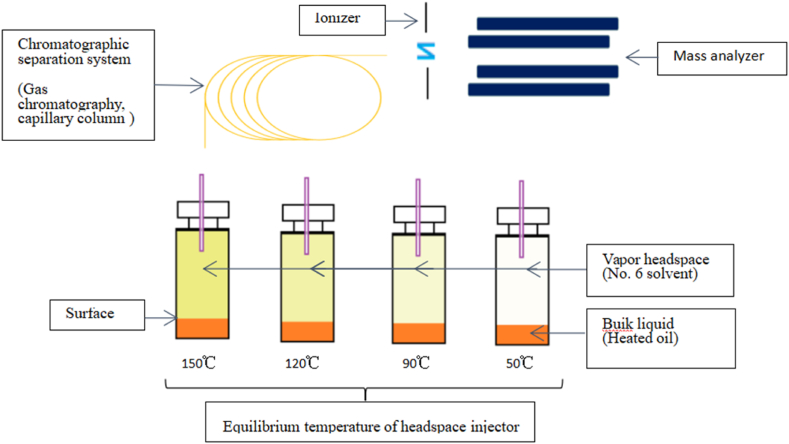
Extracted and pressed oil identification scheme by headspace coupled with GC-MS.

The conditions of the headspace sampler were as follows: sample equilibrium temperature 150 °C, balance time 30 min; sample ring 1 ml, temperature 155 °C; transmission line temperature 160 °C; pressurization pressure 138 kPa, pressurization time 0.1 min; inflation time 0.2 min; sample ring balance time 0.05 min; injection time 1.0 min. GC conditions were taken from the literature [13,20,25] and slightly modified (Fig. 2): sample inlet temperature 200 °C, HP-5MS chromatographic column (30 m × 0.25 mm × 0.25 μm); Helium (He) as the carrier gas, constant flow mode, and column flow 1 ml/min. Each sample was divided into two parts at a split ratio of 20:1. The programmed temperature rise was: 35 °C (2 min), 5 °C/min to 70 °C, 30 °C/min to 230 °C, then kept for 2 min. The mass spectrometer conditions were as follows: auxiliary interface temperature 250 °C, ion source temperature 230 °C, ionization mode ion source (EI), electron energy 70 eV, solvent delay 2.0 min, and full scan monitoring mode of 35–100 amu.

#### 2.4.2 Sample pretreatment

A vegetable oil sample (5 g) was placed in a headspace injection bottle. Then, the sealing gasket and aluminum cap of the bottle were fastened with a bottleneck opener, waiting for measurements.

#### 2.4.3 Preparation of standard solution

The No. 6 solvent was diluted to 50 μg/ml with *N*,*N*-dimethylacetamide, forming the standard solution. Seven samples were weighed, and 5 g of self-made pressed camellia seed oil was replicated in a headspace injection bottle. Then, 0, 5, 10, 20, 40, 60, and 100 μl of the No. 6 solvent stock solution was added to obtain a vegetable oil standard series with contents of 0, 0.05, 0.10, 0.20, 0.40, 0.60, and 1.00 mg/kg of No. 6 solvent respectively.

## 3 Results

### 3.1 Qualitative analysis of No. 6 solvent

Table 1 shows the five main characteristic compounds in the No. 6 solvent (Table 1).

**Table 1:** Qualitative analysis of No. 6 solvent: 5 characteristic compounds.

No.	Compound	CAS No.	Retention time/min	SIM	Quota ion
1	2-methylpentane	107-83-5	1.911	43,71,86	71
2	3-methylpentane	96-14-0	2.000	56,57,86	57
3	*n*-hexane	110-54-3	2.114	56,57,86	86
4	methylcyclopentane	1125-78-6	2.374	56,69,84	69
5	cyclohexane	110-82-7	2.748	56,69,84	84

**Fig. 3:**
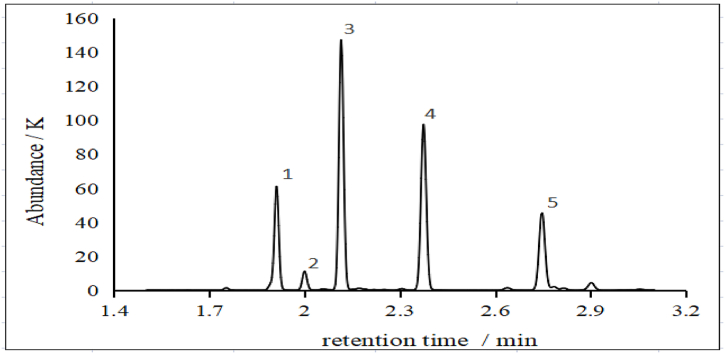
Total ion flow chromatogram of No.6 solvent standard solution (1.00 mg/kg).

Fig. 3 shows the five main peaks detected by GC in the No. 6 solvent standard solution; the peak retention times are stable. The five main peaks correspond to 2-methylpentane, 3-methylpentane, *n*-hexane, methylcyclopentane, and cyclohexane; their peak areas account for 96.6%. The standard solution also contains alkanes (e.g., 2-methylhexane, 3-methylhexane, 2,4-dimethylhexane, *n*-pentane, and *n*-heptane) and alkenes (e.g., 2-hexene and 2-ethylbutene). The total content of these minor compounds is relatively low, and their total peak area accounts for only 3.4%.

### 3.2 Residual solvent components in pressed and extracted vegetable oils

The residual solvent in the extracted *Camellia oleifera* seed oils was analyzed according to the full-scan chromatographic conditions described in Section 2.3.1. The main residual components in the No. 6 solvent were quantified according to the standard solution, and the minor components were qualitatively determined according to the highest similarity of the NIST08 chromatographic library and quantitatively analyzed using *n*-hexane as a reference (Deconinck and Jelen, 2012). The results are shown in Table 2.

**Table 2:** Residual solvents in extracted Camellia seed oils.

Peaks	Compound	Retention time, min	Content, mg/kg	CAS No.	Similarity, %
1	Pentane	1.478	0.109	000109-66-0	91
2	Pentane, 2-methyl-	1.614	0.466	000107-83-5	90
3	Pentane, 3-methyl-	1.656	2.466	000096-14-0	95
4	Hexane	1.705	8.998	000110-54-3	96
5	Pentane, 2,2-dimethyl-	1.792	0.016	000590-35-2	93
6	Pentane, methyl-	1.829	3.852	000096-37-7	91
7	Cyclohexane	1.994	0.431	000110-82-7	93
8	Hexane, 3-methyl-	2.035	0.009	000589-34-4	89
9	Heptane	2.179	0.114	000142-82-5	91
10	Cyclohexane, methyl-	2.377	0.001	000108-87-2	86
11	2-Octene, (E)-	2.925	0.001	013389-42-9	88
12	Octane	3.016	0.160	000111-65-9	87
13	p-Xylene	3.869	0.001	000106-42-3	86
14	Cyclopentane, butyl-	4.714	0.001	002040-95-1	89
15	Naphthalene	8.486	0.001	000091-20-3	91
16	Naphthalene, 1-methyl-	10.052	0.001	000090-12-0	92

Sixteen organic compounds, including pentane, 2-methylpentane, and 3-methylpentane, were detected, with a total content of 16.63 mg/kg. The 2-methylpentane, 3-methylpentane, and *n*-hexane contents accounted for 97.5%, among which *n*-hexane was the most abundant, followed by methylcyclopentane, 3-methylpentane, 2-methylpentane, and cyclohexane [13]. The total content of pentane, *n*-heptane, methylcyclohexane, and other minor components was 0.414mg/kg, accounting for 2.5% of the total, with octane being the most abundant. In addition, three aromatic hydrocarbons, including one Polycyclic aromatic hydrocarbons (PAH), were detected. Hence, the No. 6 solvent is different from the standard solution.

Although the components of the No. 6 solvent were relatively complex, five main hydrocarbon compounds were dominant, accounting for 96.6%. The five principal compounds were used as characteristic components to further identify the pressed and extracted oils.

### 3.3 Effect of headspace sampler equilibrium temperature on the peak area of characteristic components

The equilibrium temperature of the headspace sampler is the main factor affecting the peak condition of the No. 6 solvent (Drabinska et al*.*, 2012); [27]. Hence, the temperature was increased from 50 to 150 °C at intervals of 10 °C and set according to the vaporization temperature of the main components in No. 6 solvent with the lowest temperature (trimethylpentane 64 °C) and that with the highest temperature (cyclohexane 81 °C).

**Fig. 4:**
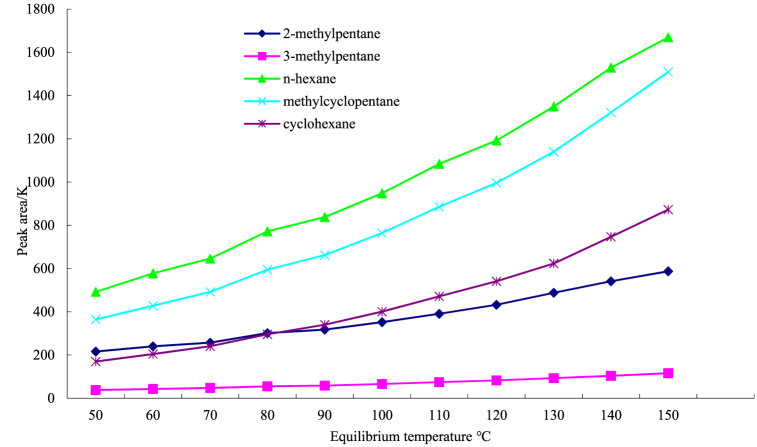
Effect of the equilibrium temperature of the headspace sampler on the peak area of No. 6 solvent.

The results are shown in Fig. 4 and illustrate that the peak area of the standard solution with a concentration of 1.00 mg/kg of No. 6 solvent increases with the equilibrium temperature. When the temperature rises to 150 °C, the peak area still increases significantly, but given the instrumental operability and method reproducibility, the headspace temperature does not continue to rise. *N*-hexane is most affected by the equilibrium temperature, with the peak area increasing by 315% from the initial to the final temperature, followed by methylcyclopentane, cyclohexane, 3-methylpentane, and 2-methylpentane. This result is related to the boiling points of the main components of the No. 6 solvent because the degree of gasification near the boiling point is significantly affected by temperature, consistent with the results reported by Ref. [13]. Consequently, the equilibrium temperature was set to 150 °C.

### 3.4 Low detection of limit (DOL) and quantitation limit (QL)

Under optimized pretreatment and chromatographic conditions, the DOL and QL of the five principal compounds of the No. 6 solvent were determined as a blank matrix and a standard solution. The standard curves were drawn with the solution concentration as the x-axis and the corresponding peak area as the y-axis (Table 3). They showed a good linear correlation over a concentration range of 0.05–1 mg/kg. The DOL was three times the signal-to-noise ratio, and the QL was set to three times the DOL. Moreover, the DOL was two orders of magnitude lower than that of the national standard (GB 5009.262-2016).

**Table 3:** Calibration curve, DOL, and QL of No.6 solvent solution.

Components	Linear equation	Correlation coefficient, R^2^	LOD, mg/kg	QL, mg/kg
2-Methylpentane	y = 583276x-578	0.999687	0.0028	0.0084
3-Methylpentane	y = 114701x-45	0.999529	0.0144	0.0433
*n*-Hexane	y = 870326x-2681	0.999785	0.0010	0.0030
Methylcyclopentane	y = 1509916x+7512	0.999438	0.0011	0.0033
Cyclohexane	y = 1682484x-960	0.999463	0.0019	0.0057

The method reported here showed a lower LOD and QL than those reported in previous studies [27]. The detection limit of the five main components in the No. 6 solvent was 0.0144 mg/kg for 3-methylpentane, and below 0.003 mg/kg for the other components. The QL of the five main components was 0.0433 mg/kg for 3-methylpentane, and lower than 0.09 mg/kg for other components.

### 3.5 Identification of pressed or extracted vegetable oils

Using the optimized conditions, the main components of the No. 6 solvent were analyzed in 42 batches of vegetable oil samples (Table 4). Except for six of self-made pressed samples, 36 batches contained No. 6 solvent as the main component. Among the five main components, *n*-hexane was detected in 36 batches, and a maximum of 8.998 mg/kg was found in oils extracted from *C. oleifera* seeds.

**Table 4:** Main components of No. 6 solvent identified in 42 batches of vegetable oil.

Vegetable oil	2-Methylpentane	3-Methylpentane	*n*-Hexane	Methylcyclopentane	Cyclohexane
	Detected sample/Sampling size (concentration, mg/kg)				
Rapeseed oil-Pressed	2/10 (0.025, 0.138)	1/10 (0.259)	10/10 (0.001–0.364)	2/10 (0.092, 0.247)	2/10 (0.081, 0.083)
Olive oil-Pressed	6/0 (ND)	6/0 (ND)	6/6 (0.001–0.003)	0/6 (ND)	0/6 (ND)
Soya-bean oil-Pressed	1/6 (0.061)	1/6 (0.367)	6/6 (0.001–0.584)	1/6 (0.614)	1/6 (0.175)
*Camellia oleifera* seed-Pressed	0/8 (ND)	0/8 (ND)	8/8 (0.001–0.003)	0/8 (ND)	0/8 (ND)
*Camellia oleifera* seed-Extracted	6/6 (0.154–0.466)	6/6 (0.287–2.466)	6/6 (1.267–8.998)	6/6 (0.742–3.852)	6/6 (0.214–0.81)
*Camellia oleifera* seed-Pressed in lab.	0/2 (ND)	0/2 (ND)	0/2 (ND)	0/2 (ND)	0/2 (ND)
Chinese torreya seeds oil-Pressed in lab.	0/2 (ND)	0/2 (ND)	0/2 (ND)	0/2 (ND)	0/2 (ND)
Chinese walnut oil-Pressed in lab.	0/2 (ND)	0/2 (ND)	0/2 (ND)	0/2 (ND)	0/2 (ND)

2-methylpentane, methylcyclopentane, and cyclohexane were detected in nine batches, reaching the limit of quantitation. The average concentrations were 0.23, 1.2, and 0.36 mg/kg; the maximum concentrations were 0.47, 3.8, and 0.81 mg/kg, respectively. 3-Methylpentane was detected in eight, with an average concentration of 0.78 mg/kg and a maximum concentration of 2.6 mg/kg. More than four main components of the No. 6 solvent were detected in nine batches simultaneously, and the total content was between 0.312 and 16.2 mg/kg. The total content of No. 6 solvent in the three batches of the pressed oil samples was 0.31–1.8 mg/kg.

*N*-hexane was the dominant solvent residue detected in all extracted oils, with a maximum of 8.998 mg/kg. Furthermore, *n*-hexane was detected in pressed oil from all industries but with a considerably lower content than that of extracted oil, possibly owing to contamination [28].

### 3.6 Determination method of pressed vegetable oils

Pressed oils are produced by mechanical pressing without chemical additives and do not contain the solvent used in the extraction method [29,30]. However, some lubricants of the squeezing machine parts contain the main components of the No. 6 solvent, as observed in a previous study [14]. Hence, during production, substances such as *n*-hexane pollute the vegetable oils and are detected in the pressed oils; however, the contents are too low to reach the quantitative limit.

A method for identifying pressed or extracted vegetable oils was established based on detecting the main components of the No. 6 solvent, using the results of the optimized detection conditions.

(1) Pressed vegetable oils: Three or fewer main components of the No. 6 solvent were detected, and the total content of the No. 6 solvent was less than 0.5 mg/kg.
(2) Extracted vegetable oils: Four or more main components of the No. 6 solvent were detected, and the total content of the No. 6 solvent was above or equal to 0.5 mg/kg.

## 4 Discussion

The headspace GC-MS method was established to identify pressed and extracted vegetable oils by analyzing the main components of the No. 6 solvent and optimizing the chromatographic conditions. This study provides a more detailed analysis and optimization procedure than a previous report [13]. No. 6 solvent is a hydrocarbon mixture mainly composed of C6, containing more than 16 types of organic substances, among which five main components, 2-methylpentane, 3-methylpentane, and *n*-hexane, account for 97%. Furthermore, the No. 6 solvent contains olefins, aromatic hydrocarbons, and polycyclic aromatic hydrocarbons, the total amount of which accounts for approximately 3%.

• The equilibrium temperature of the headspace sampler is the main factor affecting the peak area of the No. 6 solvent [31]. With increasing equilibrium temperature, the peak area was enlarged and maximized at 150 °C. Of the five main components of the No. 6 solvent, *n*-hexane was the most affected by the equilibrium temperature, and the peak area increased by 315% from the initial to the final temperatures, followed by methylcyclopentane, cyclohexane, 3-methylpentane, and 2-methylpentane. Under the optimized experimental conditions, the five main components in the No. 6 solvent showed good linearity over a concentration range of 0.05–1 mg/kg, with a correlation coefficient of 0.999.
• The method showed a lower LOD and QL than previous studies. The DOL of the five main components in the No. 6 solvent was 0.0144 mg/kg for 3-methylpentane and 0.010–0.028 mg/kg for the other components, much lower than the 0.005 mg/kg in a previous study by Ref. [13]. The QL of the five main components was 0.0433 mg/kg for 3-methylpentane and 0.030–0.084 mg/kg for the other components.
• No solvent was added to the pure pressed vegetable oils. However, the pressed oils were polluted with solvents from lubricants, sealing rings, or other plastic parts of the pressing machinery and equipment (Wen et al*.*, 2022). If three or fewer main components of the No. 6 solvent are detected, and the total content of the No. 6 solvent is less than 0.5 mg/kg, the vegetable oil is confirmed as a pressed oil. If four or more main components of the No. 6 solvent are detected, or the total content of the No. 6 solvent is ≥0.5 mg/kg, the vegetable oil is confirmed as an extracted oil.

## 5 Conclusions

In this study, we established an accurate method for distinguishing pressed oils from extracted oils using optimized headspace GC-MS. Further research is being conducted on the differences in the composition of pressed and extracted plant oils, such as nutrients, vitamin E, terpenes, squalene, and flavor components. Pressing and extracting are the main methods for manufacturing vegetable oils and are often used to improve oil output, accounting for over 99%. These vegetable oils can be identified by the oil extraction method described in this study. However, oil extraction methods for vegetable oil include not only pressing and extracting but also water enzymes, supercritical carbon dioxide, and other subcritical methods. Some of these oil extraction methods, such as water enzymes and supercritical carbon dioxide methods, do not require the use of organic solvents. Therefore, the method described in this article cannot determine whether oil extraction was conducted by pressing, the water enzyme method, or the supercritical carbon dioxide method; it can only determine whether it was conducted by leaching. In addition, some extracting methods, such as the subcritical method, use solvents other than the No. 6 solvent. Hence, misjudgment may occur if the principal component detection of the No. 6 solvent is used to infer the oil extraction method. Therefore, to obtain accurate results, a comprehensive analysis based on the specific situations, types, and contents of the detected substances must be conducted in actual detection and analysis.

## Author contribution statement

Yang Liu: Conceived and designed the experiments; Performed the experiments; Analyzed and interpreted the data; Wrote the paper.

Zhenlin Chai: Analyzed and interpreted the data; Contributed reagents, materials, analysis tools or data.

Haixia Yu: Conceived and designed the experiments; Performed the experiments; Analyzed and interpreted the data; Wrote the paper.

## Data availability statement

Data will be made available on request.

## Declaration of competing interest

The authors declare that they have no known competing financial interests or personal relationships that could have appeared to influence the work reported in this paper
